# Serine–Arginine Protein Kinase SRPK2 Modulates the Assembly of the Active Zone Scaffolding Protein CAST1/ERC2

**DOI:** 10.3390/cells8111333

**Published:** 2019-10-29

**Authors:** Duxan Arancibia, Matias Lira, Yocelin Cruz, Daniela P. Barrera, Carolina Montenegro-Venegas, Juan A. Godoy, Craig C. Garner, Nibaldo C. Inestrosa, Eckart D. Gundelfinger, Pedro Zamorano, Viviana I. Torres

**Affiliations:** 1 Departamento Biomédico, Facultad de Ciencias de la Salud, Universidad de Antofagasta, 1240000 Antofagasta, Chile; duxan.arancibia@uamail.cl (D.A.); matiaslira90@gmail.com (M.L.); yocelin.cruz@gmail.com (Y.C.); 2 Centro de Envejecimiento y Regeneración, Departamento de Biología Celular y Molecular, Facultad de Ciencias Biológicas, Pontificia Universidad Católica de Chile, 8331150 Santiago, Chile; dpbarrera@uc.cl (D.P.B.); jgodoz@gmail.com (J.A.G.); ninestrosa@bio.puc.cl (N.C.I.); 3 Department of Neurochemistry and Molecular Biology, Leibniz Institute for Neurobiology, D-39118 Magdeburg, Germany; cmontenev@gmail.com (C.M.-V.); gundelfi@lin-magdeburg.de (E.D.G.); 4 German Center for Neurodegenerative Diseases (DZNE), 10117 Berlin, Germany; craig.mgbster@gmail.com; 5 Centro de Excelencia en Biomedicina de Magallanes (CEBIMA), Universidad de Magallanes, 6210427 Punta Arenas, Chile; 6 Centre for Healthy Brain Ageing, School of Psychiatry, Faculty of Medicine, University of New South Wales, Sydney 2052, Australia; 7 Center for Behavioral Brain Sciences and Medical Faculty, Otto von Guericke University, D-39120 Magdeburg, Germany; 8 Instituto Antofagasta, Universidad de Antofagasta, 1240000 Antofagasta, Chile; 9 Departamento Bioquímica y Biología Molecular, Facultad de Ciencias Biológicas, Universidad de Concepción, 4070386 Concepción, Chile

**Keywords:** active zone, CAST1/ERC2, coiled-coil, self-aggregation, SRPK, synapse

## Abstract

Neurons release neurotransmitters at a specialized region of the presynaptic membrane, the active zone (AZ), where a complex meshwork of proteins organizes the release apparatus. The formation of this proteinaceous cytomatrix at the AZ (CAZ) depends on precise homo- and hetero-oligomerizations of distinct CAZ proteins. The CAZ protein CAST1/ERC2 contains four coiled-coil (CC) domains that interact with other CAZ proteins, but also promote self-assembly, which is an essential step for its integration during AZ formation. The self-assembly and synaptic recruitment of the *Drosophila* protein Bruchpilot (BRP), a partial homolog of CAST1/ERC2, is modulated by the serine-arginine protein kinase (SRPK79D). Here, we demonstrate that overexpression of the vertebrate SRPK2 regulates the self-assembly of CAST1/ERC2 in HEK293T, SH-SY5Y and HT-22 cells and the CC1 and CC4 domains are involved in this process. Moreover, the isoform SRPK2 forms a complex with CAST1/ERC2 when co-expressed in HEK293T and SH-SY5Y cells. More importantly, SRPK2 is present in brain synaptic fractions and synapses, suggesting that this protein kinase might control the level of self-aggregation of CAST1/ERC2 in synapses, and thereby modulate presynaptic assembly.

## 1. Introduction

The functionality of a protein depends on its conformational state and the modulation of it by post-translational modifications including phosphorylation, glycosylation and acetylation, among others [1]. These modifications regulate enzymatic activity, subcellular localization and interactions with other proteins. Frequently, proteins have the capacity of self-assembly or spontaneous assembly with interaction partners into multimolecular complexes, creating molecular machines essential for their biological function. Such macromolecular assemblies become critical in the formation of the synapse [2], where hundreds of proteins of the exocytotic machinery and the postsynaptic reception apparatus are required for neurotransmission.

In the central nervous system (CNS) synapses, at the postsynaptic site, it has been shown that Shank proteins can oligomerize via their C-terminal sterile alpha motif (SAM) [3,4], regulating excitatory synaptic transmission [5]. Similarly, the scaffold protein Gephyrin can induce the clustering of glycine receptors at inhibitory synapses through its self-aggregation properties [6,7]. At the presynaptic site, Piccolo and Bassoon are abundant AZ proteins that through self-aggregation and hetero-oligomerization participate in the assembly of the presynaptic exocytotic apparatus [8,9,10]. Therefore, the scaffold assembly has to be properly modulated to form a functional synapse. 

Much of the information about the regulation of synapse assembly comes from studies in invertebrate species. For example, at the neuromuscular junction (NMJ) of *Drosophila melanogaster,* the CAZ protein BRP was found to be critical for the functional assembly of T-bars, a specialization of the CAZ that modulates the efficacy of neurotransmitter release [11,12], indicating that the mechanisms regulating BRP levels in the synapse could control presynaptic efficiency. Genetic experiments suggested that a potential regulator for the levels of BRP at the T-bars is the SRPK79D [13,14]. In the absence of SRPK79D, BRP aggregates prematurely and ectopically along axons prior to reaching its final destination, and the formation of T-bars is disrupted. Interestingly, overexpression of SRPK79D also results in alterations of BRP recruitment at the synapses suggesting that SRPK79D is a fine regulator of synapse assembly in *Drosophila* [13,14]. 

BRP and CAST1/ERC2 share structural similarities mainly in the N-terminal domain [11] suggesting that CAST1/ERC2 could be a mammalian counterpart of BRP, with both of them sharing coiled-coil (CC) domains. CC domains are α-helical structures that self-assemble promoting the nucleation of large molecular complexes, resulting in the formation of multimeric and homomeric assemblies [15,16,17]. An interesting characteristic of CAST1/ERC2 is its propensity to form aggregates when expressed in heterologous cells. This characteristic has been used to study its direct binding to other proteins of the AZ. In this context, a direct interaction of CAST1/ERC2 with RIM1 [18] and Bassoon [19] has been observed when these proteins are co-expressed in HEK293T cells, suggesting that CAST1/ERC2 could be a key player in the assembly of the presynapse. CAST1/ERC2 contains four CC domains that are responsible for its self-assembly and its interaction with other AZ proteins containing CC domains, including Piccolo and Bassoon [10,19]; however, the mechanisms regulating this multimeric assembly is unknown.

Mammals express three genes for SRPK that are orthologous to *Drosophila* SRPK79D. The vertebrate SRPKs are also serine-arginine kinases described initially as regulators of constitutive and alternative splicing. SRPK1 is mainly expressed in the testis and pancreas [20] but has also been found in the brain [21], SRPK2 is expressed mainly in the brain [22,23] and SRPK3 in the muscle [24,25]. Overexpression of SRPK1 has been associated with gliomas, prostate, breast and lung cancers [26], and SRPK3 seems to play a role in muscle development [27]. The function of SRPKs in the CNS is limited. To date, SRPK2 has been associated with neuronal survival by a mechanism involving phosphorylation of the microtubule-associated protein tau [28,29,30]. More recently, SRPK2 has been implicated in the phosphorylation of δ-secretase, a lysosomal asparagine endopeptidase that processes tau and APP [31].

To determine whether mammalian SRPKs regulate CAST1/ERC2 self-aggregation, similar to the regulation of BRP by SRPK79D, we studied the ability of the vertebrate SRPK1, SRPK2 and SRPK3 isoforms to modulate the self-aggregation of CAST1/ERC2 in a heterologous cell system. We found that SRPK2 may regulate the aggregation of CAST1/ERC2 and that this regulation is mediated through modulation of the aggregation of its CC domains.

## 2. Materials and Methods

### 2.1. Antibodies

The following primary antibodies were used: mouse monoclonal anti-SRPK1 (1:500, DB Biosciences, San Jose, CA, USA), mouse monoclonal anti-SRPK2 (1:1000, BD Biosciences, San Jose, CA, USA), rabbit monoclonal anti-SRPK3 (Immunofluorescence, 1:1000, GeneTex, Irvine, CA, USA), rabbit polyclonal anti-SRPK3 (Western blot, 1:1000, Abexxa, Cambridge, UK), mouse monoclonal anti-^®^-Tubulin (1:5000, Abexxa, Cambridge, UK), rabbit polyclonal anti-GFP (Santa Cruz Biotechnology, Dallas, TX, USA), rabbit polyclonal anti-synaptophysin (1:100, MyBiosource, San Diego, CA, USA), rabbit polyclonal anti-CAST1/ERC2 (1:250, specific antiserum was produced against amino acids 107–138 of the rat protein and affinity purified in a column containing Sepharose GST-peptide), rabbit monoclonal anti-PSD95 (1:500, Sigma-Aldrich, San Louis, Missouri, MO, USA), rabbit polyclonal anti-mCherry (1:500, BioVision Inc., San Francisco, CA, USA), guinea pig polyclonal anti-Shank2 (1:500, Synaptic Systems, Goettingen, Germany), rabbit polyclonal anti-Piccolo (1:1000, Synaptic Systems, Goettingen, Germany), and rabbit polyclonal anti-protein disulfide isomerase (PDI) (1:1000, Santa Cruz Biotechnology, Dallas, TX, USA). The secondary antibodies for immunocytochemistry were anti-mouse and anti-rabbit Alexa Fluor^®^ 488 and 568, and anti-guinea pig Alexa Fluor^®^ 633. (Thermo Fisher Scientific, Waltham, Massachusetts, USA). For immunoblotting, horseradish peroxidase-conjugated antibodies were used to detect mouse and rabbit primary antibodies (1:3000, Invitrogen, Carlsbad, CA, USA).

### 2.2. Plasmid Construction

Standard molecular cloning and recombinant DNA techniques were used for plasmid construction. Lentiviral FUG-(SRPK)W expression vectors were assembled from the FUGW plasmid [32] in the following manner: SRPK1 (GenBank accession No. XM_017601678), SRPK2 (GenBank accession No. XM_027926460), and SRPK3 (GenBank accession No. XM_008773618) cDNAs were obtained by PCR using total RNA from rat brain tissue. Rat cDNAs for SRPKs were obtained by PCR, with the following oligonucleotide primers sets flanked by XhoI and XbaI restriction sites: SRPK1, FP 5′-CTCGAGTAATGGAGCGGAAAGTGCTC-3′ and RP 5´GGATCCTTAGGAGTTCAGCCAAG GAT-3´; SRPK2, FP 5´-CTCGAGTAATGTCAGTTAACTCTGAG-3´ and RP 5´-TCTAGACTAAGAA TTCAACCAAGGAT-3´; SRPK3: FP 5´-CTCGAGTAATGAGTGCCAGTGC CGGT-3´ and RP 5´-GGATCCCCACAGCTGCAGTAGGACC-3´. The pEGFP-C1 (Clontech, USA) was linearized using XhoI and XbaI sites and the SRPK1, -2 or -3 fragments inserted. The FUG-SRPK1, -2 or -3-W vectors were generated by subcloning the SRPKs sequences in the FUGW plasmid using the BsrGI and XbaI restriction sites. CAST1/ERC2 (GenBank accession No. AY_049038.1) and its coiled-coil domains CC1, -2, -3 or -4 sequences were obtained by PCR with the following oligonucleotide primers sets flanked by BsrGI and EcoRI restriction sites: CAST1/ERC2, FP 5′-ACTGGAATTCATGTACGGGAGTGCA AGA-3′ and RP 5′-ACTGTGTACACTATGCCCATATGCCCTC-3′; CC1, FP 5′-ACTGGAATTCT TGAGGCAGGTAAGAGAC-3′ and RP 5′-ACTGTG TACATCTTCCATGAATAGCATCAAG-3′; CC2, FP 5′-ACTGGAATTCTCCAAGTTTATGAAGACC-3′ and RP 5′-ACTGTGTACATGC AGAAGCTAATGAAGA-3′; CC3, FP 5′- ACTGGAA TTCTACCGTGATGAGTGTGGC-3′ and RP 5′- ACTGTGTACACTGAGAGTCAAGCTCTCA-3′; CC4, FP 5′-ACTGGAATTCAACTCACAGCA TTTG-3′ and RP 5′- ACTGTGTACATCCTCTAGCTGTTTCCTC-3′. Then, the FU-mRFP-W plasmid [33] was linearized using BsrGI and EcoRI enzymes, and CAST1/ERC2 and its CC domains were inserted to obtain the final constructs; namely FU-mRFP-CAST1/ERC2-W, FU-mRFP-CC1-W, FU-mRFP-CC2-W, FU-mRFP-CC3-W and FU-mRFP-CC4-W. 

For the biochemical studies performed in SH-SY5Y cells, the pEGFP and pEGFP-SRPK1, -2 and -3, in addition to pmRFP-CAST1/ERC2 and its CC-tagged domains were used. In order to obtain similar levels of expression in the cell lines for the mRFP-CAST1/ERC2 protein and its CC-tagged domains, the amounts of transfected plasmids used were adjusted according to their size and the specific quantity indicated in the figure legends. Furthermore, all the plasmids used were purified in a single batch to avoid differences in plasmid quality that could jeopardize transfection efficiency, using the NucleoBond^®^ Xtra Midi kit (Macherey-Nagel Inc., Bethlehem, PA, USA).

### 2.3. Cell Culture and Transfection

HEK293T, HT-22 and SH-SY5Y cell lines were cultured in DMEM (Dulbecco´s Modified Eagle´s medium—high glucose; Gibco, Waltham, MA, USA), supplemented with 10% (*v*/*v*) fetal bovine serum, 100 units/mL penicillin, and 100 μg/mL streptomycin (Thermo Fisher Scientific, Waltham, MA, USA). The cells were maintained at 37 °C in an atmosphere of 95% air and 5% CO_2_. Cells were transfected with the expression vectors indicated in the figures using Lipofectamine 2000 (Invitrogen, Carlsbad, CA, USA). Forty-eight hours after transfection, the cells were harvested for Western blot or fixed and stained, followed by fluorescence microscopy analysis. 

### 2.4. Rat Hippocampal Neuron Culture 

Primary cultures of hippocampal neurons were prepared from fetal rat brains (embryonic day 19; E19) according to the method of Kaech and Banker [34]. The brains were collected in precooled Hank’s balanced salt solution (HBSS), and then the hippocampi were carefully extracted. Cell suspensions were prepared by treating the tissue with 0.25% trypsin for 15 min at 37 °C, followed by homogenization. After dilution with neurobasal medium containing 0.5 mM GlutaMAX, B-27 supplement (Gibco, Waltham, MA, USA), 100 U/mL penicillin, and 100 μg/mL streptomycin, neurons were plated on culture dishes coated with 0.1 mg/mL poly-L-lysine (Sigma-Aldrich Corp. St. Louis, MO, USA). The neurons were incubated at 37 °C, 5% CO_2_.

### 2.5. Immunofluorescence Imaging and Quantification of Fluorescence Intensity

Cells were fixed with 4% paraformaldehyde in PBS for 10 min at room temperature, then permeabilized in 0.05% Triton X-100 in PBS and incubated with blocking solution (2% glycine, 2% BSA, 5% FBS, 50 mM NH_4_Cl in PBS pH 7.4) for 1 h at room temperature (RT). After incubation with appropriate primary (4 °C) and secondary antibodies (RT), coverslips were mounted with Vectashield solution (Vector, Burlingame, CA, USA). Images were acquired with a Zeiss Axiovert 10 epifluorescence microscope furnished with 40× and 100× Zeiss Apoplan oil immersion objectives and equipped with a Retiga-SRV camera operated with the standard QC capture software (Q-Imaging). Quantification was performed with ImageJ software (NIH, USA) using the corrected total cell fluorescence (CTCF) method, CTCF = integrated density – (area of selected cell × mean fluorescence of background readings), where CTCF is the corrected total cell fluorescence. Both the endogenous and recombinant protein levels were quantified, and the data were normalized to arbitrary units (A.U.).

### 2.6. Co-Immunoprecipitation

HEK293T cells were cotransfected with either FUG-SRPK1-W or FUG-SRPK2-W or FUG-SRPK3-W and FUmRFP-CAST1/ERC2-W plasmids. SH-SY5Y cells were transfected with either pEGFP-SRPK1 or pEGFP-SRPK2 or pEGFP-SRPK3 and pmRFP-CAST1/ERC2-W plasmids. Forty-eight hours after transfection cells were homogenized in RIPA buffer (10 mM Tris-HCl, 1 mM EDTA, 1% Triton X-100, 0.1% sodium deoxycholate, 0.1% SDS, 140 mM NaCl) (Millipore, Burlington, MA, USA) supplemented with 1 mM PMSF, 7 μg/mL Pepstatin, 5–10 μg/mL Leupeptin and 10 μg/mL Aprotinin. Samples containing 500 µg of total protein were precleared with Protein A/G Plus Agarose beads (Santa Cruz Biotechnology, Dallas, TX, USA) for 1 h at 4 °C. Then, the supernatant was incubated overnight at 4 °C with a polyclonal anti-GFP or mCherry antibody, and protein complexes were captured with Protein A/G Plus Agarose beads for 1 h at 4 °C. The beads were spun down at 2000× *g* for 2 min and washed three times with cold RIPA buffer before elution with 2× protein loading buffer.

### 2.7. Subcellular Fractionation

The forebrain from twelve-week-old female Sprague-Dawley rats was isolated and fractionated as described before with slight modifications [33,35]. Briefly, rat brains were homogenized in buffer A (5 mM HEPES buffer, pH 7.4, 1 mM MgCl_2_, 0.32 M sucrose (10 mL/g)) containing Halt protease inhibitor cocktail (Thermo Fisher Scientific, Waltham, MA, USA) at 900 rpm (ten strokes) using a Potter S homogenizer. The brain homogenate was centrifuged at 1000× *g* for 10 min, and the resulting supernatant was centrifuged at 12,000× *g* for 20 min obtaining pellet P2. P2 was resuspended in buffer A and loaded on top of a discontinuous sucrose gradient of 0.85, 1.0 and 1.2 M (3.0 mL each step in a Hitachi rotor P40ST) and centrifuged 3 h at 150,000× *g*. Synaptosomes were recovered from the 1.0/1.2 interface. To obtain the postsynaptic density (PSD) fraction, synaptosomes were added dropwise on a beaker containing 1 mM Tris-HCl, pH 8.1. Stirring was continued for another 30 min at 4 °C. After that, an equal volume of 1% Triton X-100, 0.32 M sucrose, 12 mM Tris-HCl, pH 8, was added and stirred for 15 min. Then, the lysate was centrifuged at 40,000× *g* for 45 min. The resulting pellet was resuspended in 0.32 M sucrose solution, loaded on a 0.85, 1.5 and 2.0 M sucrose step gradient (3.5 mL each step in Hitachi rotor P40ST) and centrifuged for three hours at 150,000× *g*. All steps were carried out at 4 °C. The PSD was collected from the 1.5/2.0 interface. Sucrose solutions were prepared in 5 mM Tris, pH 8.1. The homogenate, P2, synaptosomes and PSD were stored at −80 °C for subsequent immunoblot analysis.

### 2.8. Immunoblotting

The total protein content in the cell lysates was determined by the bicinchoninic acid assay (BCA) (Thermo Fisher Scientific, Waltham, MA, USA). Thirty micrograms of cell lysate was prepared in sample loading buffer 0.1 M Tris-HCl pH 6.8, 2.5% SDS, 0.002% bromophenol blue and 10% glycerol and heated at 95 °C for 5 min, resolved by SDS-PAGE and transferred to a PVDF membrane (Hybond, Amersham Biosciences, Piscataway, NJ, USA). Membranes were blocked with Tris-buffered saline (TBS) pH 7.4, containing 5% non-fat milk and 0.1% Tween-20. After incubation with the appropriate primary and secondary antibodies, Western blots were revealed by chemiluminescence (ECL, Amersham Biosciences, Piscataway, NJ, USA). To detect the CAST1/ERC2 oligomers, a native polyacrylamide gel in the absence of SDS was performed. The non-denatured (ND) samples were prepared in SDS free buffer with 0.1 M Tris-HCl pH 6.8, 0.002% bromophenol and 10% glycerol. Denatured (D) samples were prepared in loading buffer containing 2% SDS, β-mercaptoethanol and were boiled 10 min a 95 °C. Run was performed at a constant 70 V with running buffer using 25 mM Tris and 192 mM glycine (pH 8.3) without SDS. After running, the gel was transferred to a PVDF membrane in transfer buffer with 5% methanol and 0.5% SDS overnight at 4 °C at 30 V. First, membranes were probed with anti-mCherry antibody to detect CAST1/ERC2 and CC-tagged domains. Next, the membranes were stripped (mild stripping buffer: 1.5% glycine, 0.1% SDS, 1% Tween 20, pH 2.2, incubated at room temperature for 10 min), and reprobed with antibody against PDI. A protein ladder from 10 to 250 kDa was used (Cat. # 26619, ThermoFisher Scientific, Waltham, MA, USA).

### 2.9. Imaging Synaptic Proteins

Images of staining for SRPK2, Shank2 and Piccolo were acquired on a Zeiss Axio Imager A2 microscope with Cool Snap EZ camera (Visitron Systems) controlled by VisiView software (Visitron Systems GmbH) and in a confocal Leica SP8 microscope, objective 63× (Leibniz Institute for Neurobiology, Magdeburg, Germany).

### 2.10. Statistical Analysis

The values shown in the figures represent the mean ± SEM (standard error of the mean) of the results obtained from at least three independent experiments on separate cultures. Student’s *t*-test was used in studies when two experimental groups were compared. In studies with more than two experimental groups, statistical analysis was performed using analysis of variance (ANOVA) followed by Bonferroni´s post-test for multiple comparisons. Analyses were performed with GraphPad Prism 6 software (GraphPad Software, San Diego, CA, USA). Differences between experimental groups were considered statistically significant at a confidence level greater than 95% (*p* < 0.05).

### 2.11. Ethical Approval

All procedures performed in rat brains were in accordance with the ethical standards of Universidad de Antofagasta Ethics and Scientific Research Committee (CEIC) and Bioethical and Biosafety Committee of the Faculty of Biological Sciences, Pontificia Universidad Católica de Chile (Code number: 1806290020).

## 3. Results

### 3.1. Establishing a Model to Study SRPKs in Heterologous Cells

Protein dimerization and oligomerization are postulated as a mechanism of dynamic assembly of some AZ scaffolding proteins [10]. Intriguingly, when expressed in heterologous cells, Bassoon, CAST1/ERC2 and BRP tend to self-assemble into aggregates [19,36]. In neurons, these molecules must assemble specifically at sites of synaptogenesis, a process that must be strictly regulated. It was reported that BRP forms ectopic aggregates along axons of neurons lacking SRPK79D, while it usually localizes to synaptic boutons in wild-type neurons. This suggests that the protein kinase SRPK79D plays a central role in regulating the assembly, and thus, the spatial distribution of BRP at the T-bars of *Drosophila* larval neuromuscular junction [13,14]. Based on this observation, we sought to explore whether SRPKs can modulate CAST1/ERC2 self-assembly also in mammals.

**Figure 1:**
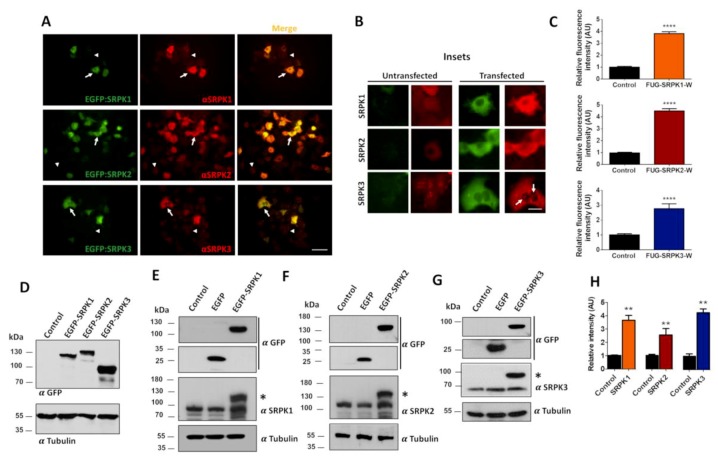
Characterization of SRPK1, -2 and -3 overexpression in HEK293T cells. (**A**). Representative images of cells transfected with pEGFP-SRPK1, -2 and -3 plasmids. Forty-eight hours after transfection cells were fixed and stained for SRPK1, -2 and -3 with the respective antibodies (red fluorescence). (**B**) The insets are a magnification of cells represented in **A,** where a diffuse pattern for the three kinases is evident, but in addition aggregates are observed for SRPK3 both in untransfected as in transfected cells. Scale bar: 5 µm (**C**) Quantification of red fluorescence intensity in non-transfected and EGFP-SRPK1, -2 and -3 transfected cells. (**D**) Western blot analysis of SRPK1, -2 and -3 overexpression. Blots were detected with anti-GFP antibody showing the respective molecular size for the EGFP-SRPK1, -2 and -3. (**E**–**G**) Western blots detected with kinase-specific antibodies comparing the expression and size of the EGFP-SRPKs with the respective endogenous kinases. The asterisks in these figures represent the EGFP-SRPK proteins. (**H**) Quantification of band intensity for the EGFP-SRPK proteins versus the endogenous kinases. A clear overexpression is observed with the transfection for the three kinases. The amounts of plasmid used for transfection were 0.5 µg/well (24-well plates, immunofluorescence) and 2.5 µg/well (six-well plates Western blot). Data are presented as mean ± SEM and statistical analysis was done by *t*-test, n = 20 cells (three independent experiments). ** *p* < 0.01; **** *p* < 0.0001. Scale bar: (**A**) 16 µm and **(B**) 5 µm.

**Figure 2:**
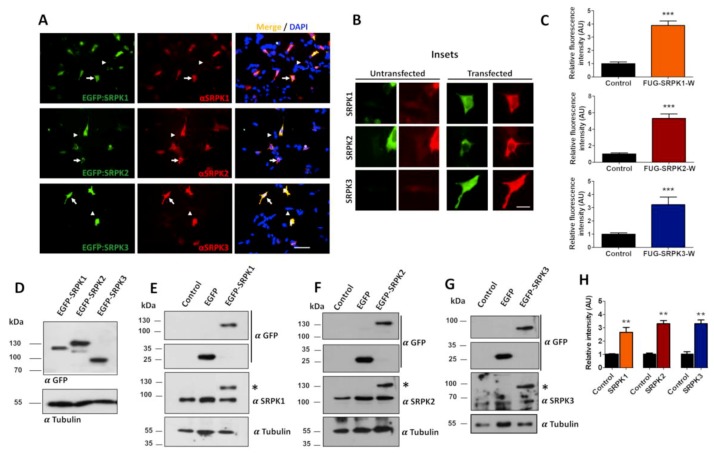
Characterization of SRPK1, -2 and -3 overexpression in SH-SY5Y cells. (**A**). Representative images of cells transfected with pEGFP-SRPK1, -2 and -3 plasmids. Forty-eight hours after transfection cells were fixed and stained for SRPK1, -2 and -3 with the respective antibodies (red fluorescence). Scale bar: 30 µm. (**B**) The insets are a magnification of cells represented in **A,** where a diffuse pattern for the three kinases is evident both in untransfected and in transfected cells. Scale bar: 10 µm. (**C**) Quantification of red fluorescence intensity in non-transfected and EGFP-SRPK1, -2 and -3 transfected cells. (**D**) Western blot analysis of SRPK1, -2 and -3 overexpression. Blots were detected with anti-GFP antibody showing the respective molecular size for the EGFP-SRPK1, -2 and -3. (**E**–**G**) Western blots were developed with kinase-specific antibodies comparing the expression and size of the EGFP-SRPKs with the respective endogenous kinases. The asterisks in these figures represent the EGFP-SRPK proteins. (**H**) Quantification of band intensity for the EGFP-SRPK proteins versus the endogenous kinases. Control bars represent endogenous SRPKs from untransfected cells, and SRPK bars represent endogenous SRPKs plus exogenous SRPKs. The amounts of plasmid used for transfection were 1.0 µg/well and (24-well plates, immunofluorescence) and 5.0 µg/well (six-well plates, Western blot). Data are presented as mean ± SEM, and statistical analysis was done by *t*-test, n = 20 cells (three independent experiments). ** *p* < 0.01; *** *p* < 0.001. Scale bar: (**A**) 30 µm and (**B**) 10 µm.

First, we tested the specificity of expression of GFP-tagged lentiviral constructs of SRPKs 1, 2 and 3 in HEK293T cells using specific antibodies for the different SRPK isoforms. As shown in Figure 1A, cells expressing the EGFP-SRPKs show high expression levels of the kinases (white arrows) in comparison with non-transfected cells (white arrowheads). The endogenous distribution of SPRK1 and SPRK2 revealed by immunofluorescence microscopy showed a diffuse expression pattern, whereas SRPK3 showed a more punctate pattern throughout the cell (untransfected cells, Figure 1B). Similarly, the recombinant EGFP-tagged kinases showed a diffuse expression pattern, with SPRK3 displaying some puncta (arrows, transfected cells, Figure 1B). Quantification of fluorescence intensity revealed a 3–4-fold increase in protein levels for all three isoforms as compared to endogenous proteins (Figure 1C). Next, we evaluated the expression levels of the kinases by immunoblot analysis. Transfected HEK293T cells expressed the correct molecular weight fusion proteins (SRPK1, 119 kDa; SRPK2, 142 kDa; and SPRK3, 89 kDa) when probed with the anti-EGFP antibody (Figure 1D). To determine SRPKs protein levels individually, blots were probed using specific SRPK1-3 antibodies. As expected, the EGFP-SPRKs transfected cells showed two immunopositive bands representing the endogenous and the EGFP-SRPKs proteins (asterisk, Figure 1E–G). Comparing the intensity of the bands of the EGFP-SRPKs with the endogenous SRPK, a 2.5–4-fold increase in SRPKs’ expression was observed (Figure 1H). Similar results can be observed in the case of SRPKs’ overexpression in the neuroblastoma cell line SH-SY5Y (Figure 2). Therefore, it is concluded that the vectors used produce overexpression of the SRPKs in both HEK293T and SHSY-5Y cell lines.

### 3.2. Effect of SRPKs on CAST1/ERC2 Aggregation in Heterologous Cells

CAST1/ERC2 and its domains CC1 and CC4 have the tendency to form homo- and hetero-oligomers [37,38,39] suggesting that they are the domains controlling CAST1/ERC2 homo-oligomerization. To investigate whether assembly mechanisms can be studied in heterologous expression systems, we sought to assess whether the SRPKs can disrupt the aggregation of CAST1/ERC2 and its CC domains. To this end, we exploited the tendency to oligomerize that CAST1/ERC2 and other CAZ proteins have when overexpressed in neurons or heterologous cells [19,39]. These oligomers are observed as large intracellular fluorescent clusters (GFP- or mRFP-tagged) [19,39] and a similar strategy was used to study the oligomerization of gephyrin [7] and alpha-synuclein [40].

**Figure 3:**
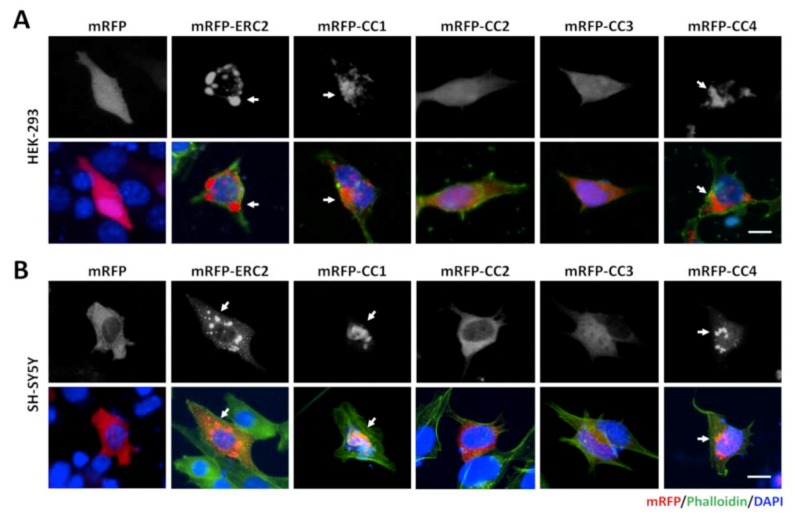
CAST1/ERC2 forms aggregate when expressed in heterologous cells. Representative images of (**A**) HEK293T and (**B**) SH-SY5Y cells transfected with mRFP, mRFP-CAST1/ERC2 or mRFP-tagged CC domains plasmids. Forty-eight hours after transfection cells were fixed and observed by fluorescent microscopy. Phalloidin conjugated with Alexa-488 was used to define cell morphology. In both cell types, CAST1/ERC2 and domains CC1 and CC4 (arrows) formed large aggregates. In contrast, CC2 and CC3 exhibited a diffuse pattern. For HEK293T cells the amounts of mRFP-ERC2, mRFP-CC1/CC2 and mRFP-CC3/CC4 plasmids used for transfection of a 24-well plate were 0.5 µg/well, 0.4 µg/well and 0.3 µg/well, respectively. For SH-SY5Y cells the amount of mRFP-ERC2, mRFP-CC1/CC2 and mRFP-CC3/CC4 plasmids used for transfection of a 24-well plate was 1.0 µg/well, 0.8 µg/well and 0.6 µg/well, respectively. Scale bars: 5 µm.

Therefore, to study the oligomerization of CAST1/ERC2, we transfected HEK293T cells with the FU-mRFP-CAST1/ERC2-W, mRFP-tagged constructs of CAST1/ERC2 coiled-coil domains (CC1–CC4) or the control mock plasmid FU-mRFP-W and analyzed the appearance of fluorescent protein cluster 48 h later. As shown in Figure S1A, the transfected cells expressed the mRFP tagged proteins at the expected molecular weight (CAST1/ERC2 (FL), 137 kDa; CC1, 47 kDa; CC2, 54 kDa; CC3, 30 kDa; and CC4, 34 kDa). In transfected HEK293T cells, expression of mRFP from the mock plasmid showed a diffuse pattern (Figure 3A), and as expected, mRFP-CAST1/ERC2 displays a cytoplasmic clustering pattern (Figure 3A, white arrows). Furthermore, the coiled-coil domains CC1 and CC4, but not CC2 or CC3, showed a prominent punctate pattern indicating self-aggregation (Figure 3A, white arrows) which agrees with the previous studies of these domains [37,38,39].

To explore whether this aggregation pattern is determined by the non-neuronal nature of HEK293T cells, we also tested the expression of these constructs in the neuroblastoma SH-SY5Y cell line. In these cells, CAST1/ERC2 and its four CC domains behaved the same way as in HEK293T cells (Figure 3B). CAST1/ERC2 and its CC1 and CC4 domains exhibited a punctate pattern, while CC2 and CC3 presented a diffuse pattern.

Next, we evaluated by Western blot the propensity of CAST1/ERC2 and its CC domains to form oligomers. To this end, HEK293T cells were transfected with the plasmids expressing full-length CAST1/ERC2 or either one of the four CC domains, all tagged with mRFP. Thirty-six hours after transfection, half of the cell lysate was mixed with denaturing sample buffer and heated at 95 °C, and the other half of the lysate was prepared in nondenaturing sample buffer (see Materials and Methods). The samples were run in a native polyacrylamide gel. In non-denaturing conditions, CAST1/ERC2 migrates more slowly in comparison with the denatured sample (Figure S1B) indicating the formation of oligomers. CC2 and CC3 domains showed the same migration behavior under both denaturing and non-denaturing conditions, suggesting that these recombinant proteins do not oligomerize (Figure S1C). On the other hand, a more slowly migrating band was observed in the undenatured samples for CC1 and CC4 domains suggesting the formation of oligomers for these constructs. The cytosolic soluble protein disulfide isomerase (PDI), which was used as a control of a non-oligomerizing protein, showed the same migration in both conditions (Figure S1B,C). As in native gels polypeptides do not migrate exactly according to their size, but rather migration is significantly influenced by the proteins’ conformations, the exact size of the oligomers could not be determined. However, the results of Figure S1B,C support the findings of Figure 3, i.e., mRFP-ERC2 forms oligomers in the cell, and this seems to be mediated by CC1 and CC4 domains.

**Figure 4:**
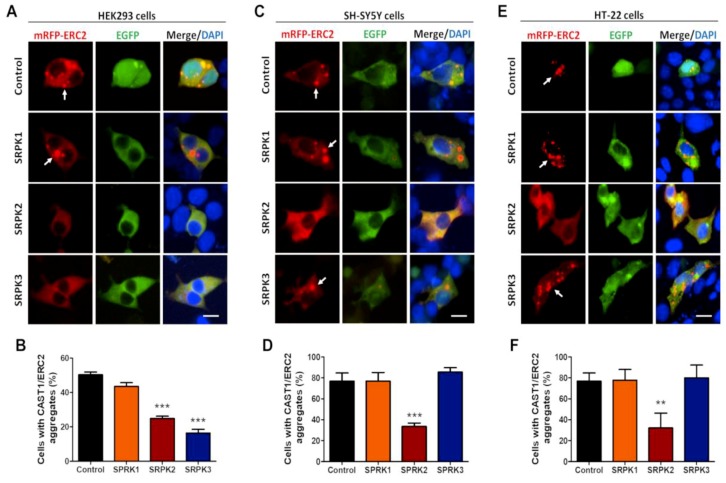
SRPK2 disrupts the aggregation of CAST1/ERC2 in heterologous cells. (**A**) HEK293T, (**C**) SH-SY5Y and (**E**) HT-22 cells were co-transfected with mRFP-CAST1/ERC2 and EGFP (control) or EGFP-SRPK1, -2 and -3 plasmids. Graphs show the quantification of the percentage of cells containing mRFP-CAST1/ERC2 aggregates in (**B**) HEK293T, (**D**) SH-SY5Y and (**F**) HT-22 cells. For mRFP-CAST1/ERC2 and EGFP-SRPKs the plasmid amount used for transfection of a 24-well plate was 0.5 µg/well for HEK293 cells, and 1.0 µg/well for SH-SY5Y and HT-22 cells. Data are presented as mean ± SEM, and statistical analysis was done by ANOVA and Bonferroni’s post-hoc test; n = 100 cells (three independent experiments). ** *p* < 0.01; *** *p* < 0.001. Scale bar: 15 µm.

Next, to explore if the SRPKs control the state of aggregation of CAST1/ERC2, recombinant mRFP-CAST1/ERC2 and EGFP-SRPK1, -2, or -3 were co-expressed in HEK293T cells, using EGFP expression as a mock control. As expected, mRFP-CAST1/ERC2 formed large aggregates in ~50% of transfected cells (white arrows, Figure 4A,B; 50.3% ± 1.40%). In cells co-transfected with SRPK2 (24.8% ± 1.32%) and SRPK3 (16.4% ± 2.20%), the fraction of cells with CAST1/ERC2 aggregates was significantly reduced (Figure 4A,B). However, even in cells expressing SRPK1, CAST1/ERC2 aggregates still were formed (Figure 4A,B; 43.5% ± 1.46%). As mentioned before, HEK293T cells have been used as a tool to study CAST1/ERC2 self-aggregation and protein interactions. To verify if the same mechanisms act in neuron-like cells, the oligomerization of CAST1/ERC2 was also studied in the human neuroblastoma SH-SY5Y and the mouse hippocampal HT-22 cell lines.

Similar to HEK293T cells, CAST1/ERC2 showed a punctate pattern in nearly 80% of the transfected SH-SY5Y and HT-22 cells (Figure 4C,E, respectively). Interestingly, only SRPK2 was able to reduce the fraction of cells with CAST1/ERC2 aggregates in both neuronal cell lines (SH-SY5Y, 33.65% ± 3.18%; HT-22, 32.14% ± 14.14%) (Figure 4D,F, respectively), suggesting that the neuronal environment favors a more specific role of SPRK2 in CAST1/ERC2 disaggregation.

### 3.3. SRPKs Affect Self-Aggregation of Coiled-Coil Domains 1 and 4 of CAST1/ERC2

**Figure 5:**
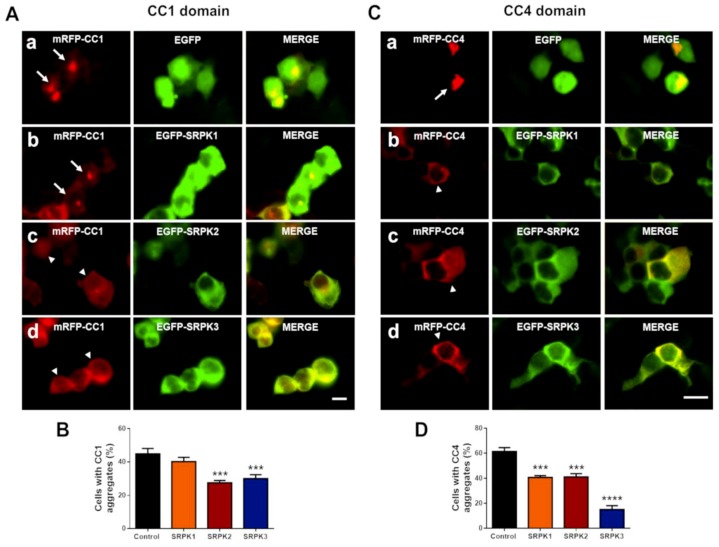
SRPKs disrupts the aggregation CC1 and CC4 domains of CAST1/ERC2 in HEK293T. Representative images of HEK293T cells co-transfected with either EGFP (control) or EGFP-SRPK1, -2, -3 and mRFP-tagged (**A**) CC1 and (**C**) CC4 domains plasmids. Forty-eight hours later, the cells were fixed and analyzed by fluorescence microscopy. Graphs show the quantification of the percentage of cells containing (**B**) CC1 and (**D**) CC4 aggregates. The plasmid amounts used for transfection of a 24-well plate were 0.4 and 0.3 µg/well for CC1 and CC4, respectively. In the case of SRPKs the plasmid amount used for transfection was 0.5 µg/well. Data are presented as mean ± SEM, and statistical analysis was done by ANOVA and Bonferroni’s post-hoc test; n = 100 cells (three independent experiments). *** *p* < 0.001; **** *p* < 0.0001. Errors bars represent SD. Scale bar: 10 µm.

**Figure 6:**
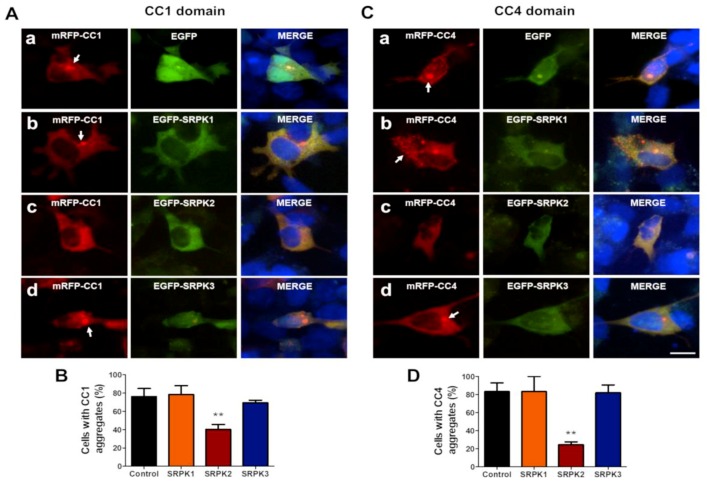
SRPK2 specifically disrupts the aggregation of CAST1/ERC2 domains CC1 and CC4 in the neuroblastoma SH-SY5Y. Representative images of SH-SY5Y cells co-transfected with either EGFP (control) or EGFP-SRPK1, -2, -3 and mRFP-tagged (**A**) CC1 and (**C**) CC4 domain plasmids. Forty-eight hours later, the cells were fixed and analyzed by fluorescence microscopy. Graphs show the quantification of the percentage of cells containing (**B**) CC1 and (**D**) CC4 aggregates. The plasmid amounts used for transfection of a 24-well plate were 0.8 and 0.6 µg/well for CC1 and CC4, respectively. In the case of SRPKs the plasmid amount used for transfection was 1.0 µg/well. Data are presented as mean ± SEM and statistical analysis was done by ANOVA and Bonferroni’s post-hoc test; n = 100 cells. ** *p* < 0.01 (three independent experiments). Errors bars represent SD. Scale bar: 10 µm.

To study whether co-expression of SRPKs induces disaggregation of CC1 and CC4 domains, HEK293T cells were co-transfected either with FU-mRFP-CC1 or CC4 and EGFP-SRPK1, -2 or -3. As expected, the expression of CC1 (Figure 5(Aa,B), white arrows; 44.87% ± 3.21%) and CC4 (Figure 5(Ca,D), white arrows; 61.41% ± 3.93%) led to the formation of intracellular aggregates. Interestingly, both SRPK2 (Figure 5(Ac), white arrowhead; 27.56% ± 1.39%) and SRPK3 (Figure 5(Ad), white arrowhead; 30.13% ± 2.29%), but not SRPK1 (Figure 5(Ab); 35.54% ± 1.58%), significantly decreased the number of cells with CC1 aggregates (Figure 5B). In the case of CC4, all three kinases significantly reduced the number of cells with CC4 aggregates (Figure 5(Cb,c,d,D); 40.85% ± 1.39%; 41.27% ± 2.57%; 15.35% ± 2.41%, respectively), as is shown by white arrowheads. Interestingly, SRPK3 overexpression had no significant effect on the aggregate formation of CAST1/ERC2 in the neuron-like cell lines (Figure 4C,E). Therefore, we next tested whether SRPKs could act on the state of aggregation of CC1 and CC4 domains when these are expressed in SH-SY5Y cells. Again, in this neuron-like cell line, only SRPK2 was able to reduce the number of cells containing CC1 (Figure 6A,B) and CC4 (Figure 5C,D) aggregates. This suggests that SRPK2 is the main regulator of CAST1/ERC2 oligomerization in neuron-like cells.

### 3.4. Exploring Whether SRPK2 Forms a Complex with CAST1/ERC2

**Figure 7:**
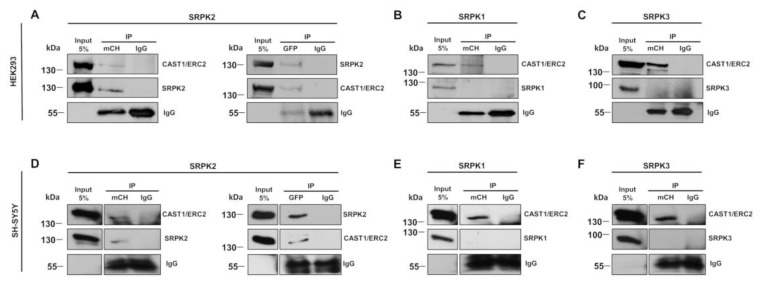
CAST1/ERC2 interacts with SRPK2 in HEK293T and SH-SY5Y cells. HEK293T and SH-SY5Y cells were co-transfected with EGFP-SRPK1, EGFP-SRPK2 or EGFP-SRPK3 and mRFP-CAST1/ERC2 plasmids. Forty-eight hours later, cells were lysed and immunoprecipitated with GFP or mCherry antibody. Normal rabbit IgG was used as a control. Immunoprecipitated proteins were analyzed by Western blotting using mCherry or GFP antibodies, both in (**A**) SRPK2, (**B**) SRPK1 and (**C**) SRPK3 in HEK293T and (**D**) SRPK2, (**E**) SRPK1 and (**F**) SRPK3 in SH-SY5Y cells. For mRFP-ERC2 and EGFP-SRPKs the amounts of plasmid used for transfection of a six-well plate were 2.5 µg/well and 5.0 µg/well for HEK293T and SH-SY5Y cells, respectively. Images represent three independent experiments.

Since SRPK2 expression is highly detected in the brain [22,23] and based on data reported above, i.e., that SRPK2 acts specifically on CAST1/ERC2 aggregates, we wondered if SRPK2 interacts directly with CAST1/ERC2. To this end, co-immunoprecipitation experiments were performed after co-expressing mRFP-CAST1/ERC2 full-length and EGFP-SRPK2 in HEK293T and SH-SY5Y cells. Reciprocal immunoprecipitation with mRFP and GFP antibodies to detect CAST1/ERC2 and SRPKs interactions were performed. Immunoblot analysis revealed that SRPK2 and CAST1/ERC2 proteins co-immunoprecipitated (Figure 7A,D). Also, the interactions between SRPK1 (Figure 7B,E) and SRPK3 (Figure 7C,F) with CAST1/ERC2 were tested. The results show that SRPK1 and SRPK3 do not co-immunoprecipitate with CAST1/ERC2, suggesting that the interaction between SPRK2 and CAST1/ERC2 is specific. Although this experiment does not show a direct association between SRPK2 and CAST1/ERC2, it does indicate that they can exist in the same protein complex.

### 3.5. SRPK2 Is Localized in Synapses of Hippocampal Neurons in Culture

**Figure 8:**
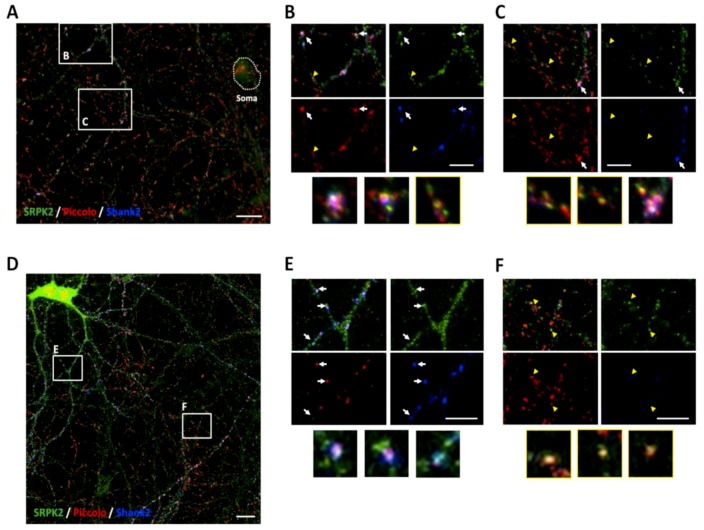
SRPK2 colocalizes with synaptic proteins in cultured hippocampal neurons. Representative images show neurites in dissociated rat hippocampal cultures. Neurons 21 DIV were triple stained using anti-SRPK2, anti-Piccolo and anti-Shank2 antibodies. Arrows indicate the co-distribution signal of SRPK2 with both Piccolo and Shank2, and arrowheads indicate co-distribution of SRPK2 only with Piccolo. (**A**) Low magnification and (**B**,**C**) high magnification of images acquired in an epifluorescence microscope. (**D**) Confocal images showing colocalization of SRPK2 with Piccolo and Shank2 (arrows), and only with Piccolo (arrowheads). (**E**,**F**) Digital magnifications of colocalization sites from (**D**). Scale bar: (**A**,**D**) 20 µm and (**B**,**C**,**E**,**F**) 10 µm.

Taken together, our results suggest a role of SRPK2 on CAST1/ERC2 self-assembly, yet whether this function is at play within synapses is unknown. Hence, we evaluate the presence of SRPK2 in synapses by two approaches. First, hippocampal neurons 21 days in vitro (DIV) were fixed and immunostained for the presynaptic protein Piccolo, postsynaptic protein Shank2 and SRPK2. Acquisition by fluorescence microscopy revealed partial co-distribution of SRPK2 with Piccolo and Shank2 (Figure 8A; arrows, insets B and C) (and Bassoon, not shown) and co-distribution only with Piccolo in neurites (Figure 8A; arrowheads, insets B and C). A more detailed analysis by confocal microscopy showed similar results. Here, SRPK2 co-localized with both synaptic markers (Figure 8D arrows) and also some puncta co-localized only with Piccolo (Figure 8D arrowheads). In Figure 8E, digital magnifications of colocalization sites (arrows) from Figure 8D are shown. Colocalization studies with CAST1/ERC2 were not possible since the antibody for CAST1/ERC2 was not suitable for immunocytochemistry; however, at this stage in the neuronal cultures (21 DIV) this protein is primarily synaptic [18].

**Figure 9:**
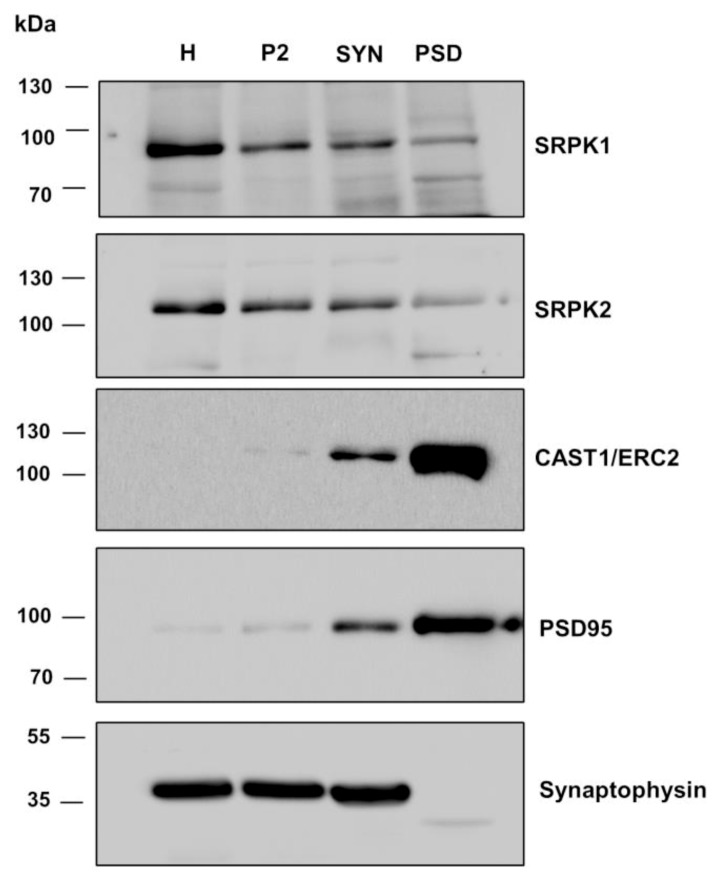
Endogenous SRPK1 and SRPK2 associate with synaptic brain fractions. Adult rat brains were homogenized and fractionated (see methods). Thirty micrograms of protein of each fraction were fractionated by SDS-PAGE. Western blots were probed with antibodies against SRPK1, SRPK2, SRPK3, CAST1/ERC2, synaptophysin and PSD95. PVDF membranes were stripped and reprobed to analyze all proteins. Molecular-weight size markers standards are shown on the left. H, homogenate; P2, crude membranes; Syn, synaptosomes; PSD, postsynaptic density (images represent three independent experiments).

In a second strategy, we performed biochemical fractionation experiments of brain homogenates and studied the distribution of the three kinases in synaptic compartments. Here, we used a well-established protocol (Materials and Methods) to isolate P2 containing crude membranes (comprised of synaptosomes, myelin, light membranes, and mitochondria) and synaptosomes which contain synaptic plasma membranes, PSD and synaptic vesicles. Further biochemical fractionation of synaptosomes led to the isolation of the PSD fraction. This contains the synaptic cytoskeleton and proteins tightly associated with synaptic junctions, including components of the postsynaptic density and the presynaptic CAZ [41]. As expected, the marker protein PSD95, a major scaffolding protein of the postsynaptic apparatus, and CAST1/ERC2 (Figure 9), as well as Bassoon and Piccolo (data not shown), were highly enriched in synaptosomes and PSD fractions as compared with the homogenate. Synaptophysin, a synaptic vesicle protein, was highly enriched in synaptosomes which contain synaptic vesicles but was excluded from synaptic junctional preparations (PSD fraction, Figure 9). Hence, the distribution and enrichment of those proteins validate the biochemical preparations of this work. SRPK1 and 2 were present in synaptosomes and the PSD fractions, though they were not enriched when compared with the homogenate (Figure 9). Nonetheless, it can be concluded that these two proteins are present at synapses. The PSD fraction does not contain lipid membranes, which were removed with Triton X-100 treatment, suggesting that a fraction of SRPK1 and SRPK2 could be tightly associated to the synaptic cytomatrix (i.e., CAZ and/or PSD). We could not detect a signal for SRPK3 in the brain (data not shown); this has been reported to be specifically expressed in muscle [24]. Altogether these data suggest that SRPK1 and SPRK2 are present in synapses and that SRPK2 might modulate the self-assembly of CAST1/ERC2 within presynaptic terminals.

## 4. Discussion

During synaptogenesis, presynaptic AZ components traffic along axons in preassembled vesicular complexes that require further molecular organization prior to becoming part of a functional presynaptic site [42]. It is thought that local signals present at nascent synapses might play an important role in the recruitment and/or trapping of AZ components, through cell adhesion molecules such as neuroligins, SynCAM, neuroplastin, among others, which act to specify nascent synaptic sites [43,44]. Piccolo and Bassoon are known to form homo- and heterodimers with other AZ proteins such as CAST1/ERC2 through CC domains [18,19] and are thought to be central organizers of the CAZ. These proteins, if overexpressed in neurons or heterologous cells, tend to form large intracellular aggregates [19,45], indicating that to correctly assemble at synapses, their local expression levels, conformational state and/or trafficking towards newly presynaptic sites must be finely regulated. The molecular mechanisms governing these processes for most of the presynaptic proteins are unknown. Here, we provide some evidence that mammalian SRPKs might be part of the signaling mechanism regulating the assembly of CAST1/ERC2 at CAZs.

For the BRP protein, it was shown that the kinase SRPK79D is necessary for the correct spatiotemporal assembly of the T-bars from the *Drosophila* neuromuscular junction [13,14]. In the present study, we have examined whether mammalian SRPK family members may similarly modulate the clustering of CAST1/ERC2 using a heterologous expression system. Ko et al. [36] and Takao-Rikitsu et al. [19] utilized the aggregation properties of CAST1/ERC2 protein as a co-clustering assay to determine its association with Syntenin-1 and other AZ proteins. In our studies, we used the aggregation property of CAST1/ERC2 to explore whether the co-expression of different mammalian SRPKs can modulate the clustering CAST1/ERC2 in heterologous cells.

Intriguingly, we observed that SRPK2 and SRPK3 inhibited CAST1/ERC2 aggregation in HEK293T cells. More importantly, when these experiments were repeated in SH-SY5Y and HT-22 cells lines, similar results were obtained for SRPK2, significantly reducing the CAST1/ERC2 aggregates, while SRPK1 and SRPK3 do not affect the aggregation of CAST1/ERC2. It is worth mentioning that SRPK3 is specifically expressed in the heart and muscle [24,25], while SRPK2 is highly expressed in the brain [22,23]. It is thus possible that in these neuronal cell lines, an unknown mechanism could prevent SRPK3 from exerting any action on the clustering of CAST1/ERC2. Nonetheless, these experiments indicate that SRPK2 could be an important regulator of CAST1/ERC2 oligomerization.

As both the CC1 and CC4 domains have been shown to interact and form hetero-oligomers with full-length CAST2/ERC1b [39], we also examined whether the oligomerization of CC1 and CC4 could be modulated by SRPK1, -2 and -3 in these cell lines. Similarly to the effects observed in the full-length molecule, SRPK2 and SRPK3 modulate the aggregation of CC1 and CC4 in HEK293T cells. Interestingly, the effect was also observed in CC4 aggregation by SRPK1. However, the inhibition of the CC1 and CC4 aggregation in SH-SY5Y cell is only accomplished by SRPK2 overexpression. These data suggest that the control of CC1 and CC4 oligomerization depends on the protein context, as SRPK1 does not affect the full-length CAST1/ERC2 oligomerization, but also depends on the cellular context, as only SRPK2 modulates CC1 and CC4 aggregation in SH-SY5Y and HT-22 cells. The fact that SRPK2 is the main SRPK expressed in the brain and modulates the aggregation of CAST1/ERC2 and the CC1 and CC4 in neuron-like cell lines would indicate that SRPK1 and SRPK3 may not be relevant in a neuronal context.

In order to better characterize the cluster observed in the heterologous cell systems, we performed oligomerization analysis by fractionation of protein assembles in native PAGE Western blot. The results showed that CAST1/ERC2 and CC1 and CC4 domains form homo-oligomers in HEK293T cells, demonstrating that the fluorescent clusters observed were formed by an oligomerization state of these proteins. Furthermore, immunoprecipitation analysis showed that SRPK2 is the only SRPK that interacts with CAST1/ERC2; although this assay does not demonstrate a direct interaction, it does provide some evidence that both proteins are present in a molecular complex, although further studies are required to determine whether this interaction is direct.

SRPK79D is found at synapses colocalizing with Bruchpilot in the invertebrate synapse [14]. We also found by subcellular fractionation that SRPK1 and SRPK2 are part of the PSD. The latter suggests a strong association, as the PSD is a detergent-treated subcellular fraction. Notably, we could detect SRPK2 immunoreactivity at glutamatergic synapses, strongly suggesting that the SRPK2 is part of the synaptic apparatus.

Direct phosphorylation of CAST1/ERC2 at serine 45 by serine-threonine kinase SAD-B has been shown to induce a decrease in the number of synaptic vesicles clustered presynaptically within the readily releasable pool of SVs, suggesting a role in synaptic efficacy [46]. At present, it is unclear how SRPK may influence AZ assembly or synaptic vesicle release though CAST1/ERC2. Driller et al. showed that SRPK79D phosphorylates BRP in its amino-terminal, allowing its normal axonal transport [47]. In the same study using an in vitro assay, SRPK1 phosphorylated an N-terminal fragment of CAST2. As we found SRPK2 in synapses, conceptually, one would anticipate that SRPK2 may control the assembly and/or synaptic function through the phosphorylation of CAST1/ERC2, although phosphorylation-independent actions for SRPK2 have also been described [48]. Further studies are required to demonstrate phosphorylation of CAST1/ERC2 by SRPK2.

Together with published data, SRPKs appear to be kinases with versatile roles in neurons, including RNA splicing [23], the regulation of the assembly of tubulin through phosphorylation of tau [29], and now their presence in synaptic junctions, where they could be strategically positioned to modulate the dynamic assembly of CAST1/ERC2 along with other AZ scaffolding proteins during synapse formation and function.

## 5. Conclusions

We presented evidence that SRPK2 can regulate the self-assembly of CAST1/ERC2 and this oligomerization is regulated through CC1 and CC4 domains. Our findings further showed that these two proteins are part of a complex and that SRPK2 is enriched in synaptosomes and synaptic junctions. As such SRPK2 could an important regulator of synapse assembly and function.

## Supplementary Materials

The following are available online at https://www.mdpi.com/2073-4409/8/11/1333/s1.
