# Supplementary material for: Serine–Arginine Protein Kinase SRPK2 Modulates the Assembly of the Active Zone Scaffolding Protein CAST1/ERC2
Source: Cells. 2019 Oct 29;8(11):1333. doi: 10.3390/cells8111333 (PMC6912806; doi:10.3390/cells8111333)
Supplement: Supplementary file 1 [file cells-08-01333-s001.pdf]

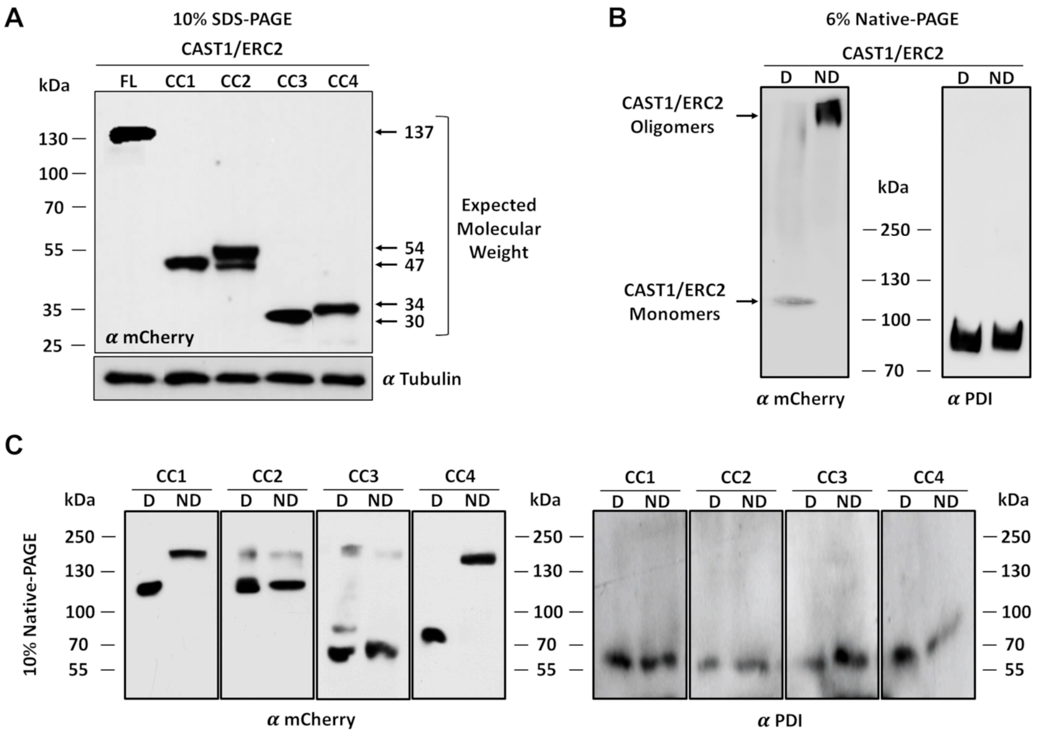

Figure S1. Expression of recombinant full-length CAST1/ERC2 protein and individual coiled-coil domains in HEK293T cells, and analysis of oligomerization under non-denaturing conditions. (**A**) mRFP-CAST1/ERC2 (FL) and its mRFP-tagged CC domains were expressed in HEK293T cells. Detection of mRFP-tagged protein in the cell lysates was performed using anti-mCherry antibody as indicated in Material and Methods. FL: Full-length mRFP-CAST1-ERC2; CC: coiled-coil domains 1, 2, 3 and, 4. On the left migration positions of size markers are indicated, on the right side the calculated sizes for each mRFP-tagged fusion protein is shown. All constructs displayed the correct size. In CC2 a lower molecular weight band was observed, probably due to cleavage or partial degradation. (**B**) Western blot analysis of 6% native gel loaded with mRFP-CAST1/ERC2, either denatured (**D**) by treatment with denaturing buffer or non-denatured (ND), i.e. cell lysate was treated with non-denaturing buffer (see Material and methods). (**C**) Western blot analysis of 10% native gels of mRFP-CC1, CC2, CC3, or CC4 under denaturing or non-denaturing conditions. In **B and C**, blots were stripped and reprobed for the cytosolic soluble protein disulfide isomerase (PDI), which was used as an example of a non-oligomerizing protein and showed the same migration in both conditions. The CC1 and CC4 domains showed different migrations patterns of under non-denaturing and denaturing conditions, while the CC2 and CC3 present the same migration pattern independent of the denaturing treatment. Positions of molecular-weight size markers are indicated. The amounts of plasmid used for transfection of a 6-well plate were 2.5 μg/well for CAST/ERC2; 2.0 μg/well for CC1 and CC2 domains; and 1.5 μg/well for CC3 and CC4 domains.
